# MptpB Promotes Mycobacteria Survival by Inhibiting the Expression of Inflammatory Mediators and Cell Apoptosis in Macrophages

**DOI:** 10.3389/fcimb.2018.00171

**Published:** 2018-05-25

**Authors:** Lingbo Fan, Xiaoyu Wu, Chunyan Jin, Fengge Li, Sidong Xiong, Yuanshu Dong

**Affiliations:** Jiangsu Key Laboratory of Infection and Immunity, Institute of Biology and Medical Sciences, Suzhou, China

**Keywords:** MPtpB, TB, macrophage, apoptosis, inflammation

## Abstract

Tuberculosis is a severe contagious disease caused by *Mycobacterium tuberculosis* (Mtb). To develop new vaccines and medicine against TB, there is an urgent need to provide insights into the mechanisms by which Mtb induces tuberculosis. In this study, we found that secreted Mtb virulence factor MptpB significantly enhanced the survival of H37Rv in macrophages. MptpB suppressed the production of iNOS, the expression of inflammatory factors IL-1β and IL-6, as well as the apoptosis of the macrophage in Mtb infected RAW264.7 cells. Mechanism investigation showed that MptpB simultaneously hampered the NF-κB and MAPK signal pathways, evidenced by its blocking of p65, IKKα, Erk1/2, and p38 phosphorylation induced by Mtb infection. MptpB also inhibited host cell p53 expression. The results demonstrated that MptpB contributed to the survival of H37Rv by inhibiting host inflammatory responses and apoptosis through impeding the NF-κB and MAPK signal pathways and p53 expression in the macrophage.

## Introduction

Tuberculosis is a chronic contagious disease caused by *Mycobacterium tuberculosis* that threatens human life. Mtb infected one third of the world's population, resulted in 6.1 million new patients and 1.4 million deaths in 2015 (www.who.int). The treatment against active TB needs more than 6 months, which often leads to multidrug-resistant strains of mycobacterium tuberculosis (MDR-TB) due to inadequate treatment and poor patient compliance (Esmail et al., 2014; Seung et al., 2015; Dheda et al., 2017; Imperiale et al., 2017). MDR-TB has become a major worldwide threat to the public currently, and about half of MDR-TB patients were not successfully treated. Therefore, it is necessary for us to explore the molecular mechanisms of interplay between *M. tuberculosis* and the host immune system to identify novel effective therapeutic targets.

Macrophages serve as the first line of host immune systems against Mtb and play a major role in determining tuberculosis development (Cambier et al., 2014; Song et al., 2015; Li et al., 2017). Mtb elicits numerous immune evasion mechanisms to favor its survival and proliferation and eventually results in formation of tuberculosis (Stokes and Waddell, 2009). Several such mechanisms have been discovered, including inhibiting the formation of phagolysosome, avoiding the toxic effects of reactive nitrogen species, affecting cell proliferation or migration, and interfering with antigen presentation (Kang et al., 2005; Reusch et al., 2006; Vandal et al., 2009; Torrelles and Schlesinger, 2010; Liu et al., 2017). Our study on the mechanisms how Mtb virulence factor MptpB induces immune evasion will provide basis for a better treatment strategy of tuberculosis.

MptpB is a critical virulence factor secreted by Mtb. Its structural information indicates that it belongs to the family of protein tyrosine phosphatase (He et al., 2015; Ghattas et al., 2016), whose protein conformation switches between “closed” and “open” forms to resist oxidative inactivation (Flynn et al., 2010). *In vitro* experiments showed that it has triple substrate specificity toward phosphortyrosine, phosphorserine/threonine, and phosphoinositides (Beresford et al., 2007), and its activity appears to be regulated by lysine acylation (Singhal et al., 2015). Mtb secretes two protein tyrosine phosphatases, MptpA and MptpB (Wong et al., 2013). MptpA deficiency resulted in decreased survival of BCG but not the virulent strain Erdman in mice (Grundner et al., 2008; Wang et al., 2015). Disruption of MptpB in Erdman led to a 70-fold reduction of bacterial burden in guinea pigs (Singh et al., 2003) and a 5–7-fold reduction in activated macrophages (Singh et al., 2003; Chauhan et al., 2013), indicating that MptpB impairs the antimicrobial ability of activated macrophages and serves as a promising therapeutic target against Mtb. Several inhibitors aimed at the phosphatase active site of MptpB had been developed, whose effects on eliminating Mtb survival in macrophage were quite mild, not comparable to that of MptpB deficiency (Soellner et al., 2007; Zhou et al., 2010), suggesting that the mechanisms how MptpB affects the functions of macrophage needs to be further explored. In this research, we overexpressed MptpB in RAW264.7 cells and found that it significantly increased the survival of mycobacteria H37Rv in macrophages. The results showed that MptpB inhibited the expression of proinflammatory cytokines and the apoptosis of macrophages induced by H37Rv infection, therefore led to increased bacillary load. We also proved that MptpB hampered the bactericidal responses of macrophages by inhibiting NF-κB and MAPK signal pathways.

## Materials and methods

### Bacterial strains and culture conditions

BCG was obtained from the Center for Disease Control of Suzhou. *Mycobacterium tuberculosis* H37Rv and *Mycobacterium smegmatis* mc^2^155 were from Reference Lab of TB control center in Guangdong province. Mycobacteria were cultured in Middlebrook 7H9 broth (BD) supplemented with 10% Middlebrook OADC enrichment (BD), 0.5% glycerol as well as 0.05% Tween 80. Mtb cultures were handled under the specific biosafety recommendations established by WHO (2012). All protocols were approved by the Institutional Biosafety Committee of Soochow University.

### Cell culture and mycobacterial infection

RAW264.7 was cultured with DMEM supplemented with 10% Fetal Bovine Serum (Gibco, Australia), 0.1 mg/mL streptomycin, 100 U/mL penicillin and 10 mM glutamine (Invitrogen) with 5% CO2 at 37°C. LPS was from Sigma. Pam3CSK4 was from InvivoGen. Murine IFN-γ was from PeproTech. Unless otherwise specified, RAW264.7-MptpB cells (5 × 10^5^) for western blot and QPCR were infected with H37Rv at MOI = 10 for 6 h whereas RAW264.7 cells (1 × 10^5^) for detecting CFU were infected with 1 × 10^6^ CFUs of Mtb. The infected RAW264.7 was collected at 0, 2, 4, and 6 days. Individual colonies were counted to assess CFUs by growing for 3 weeks at 37°C.

### Plasmid construction

MptpB was amplified from H37Rv genomic DNA and sub-cloned into prokaryotic expression vector pET28b, eukaryotic expression vector pFLAG-cmv2 or retroviral expression vector pMSCV-eGFP to construct the plasmid pET28b-MptpB, pFLAG-cmv2-MptpB or pMSCV-eGFP-MptpB, respectively. Catalytically inactive C106S mutant of MptpB (MptpB/CS) expression vectors were also constructed using Quikchange Site-Directed Mutagenesis Kit (Stratagene). FLAG sequence is 5′-GACTACAAAGACGATGACGACAAG-3′. Both His-tag (in pET28b) and FLAG-tag (in pFLAG-cmv2 and pMSCV-eGFP) were cloned to the N-terminal of MptpB.

### Expression of MptpB in *Escherichia coli*

Rosetta strain was transformed with pET28b-MptpB, and the expression of His-MptpB protein was induced with 0.5 mM isopropyl β-D-1-thiogalactopyranoside (IPTG) (Beyotime, Jiangsu, China) at 25°C overnight. His-MptpB protein was then purified using Ni-NTA His-binding resin (GE healthcare) and eluted with 100 mM imidazole. LPS in purified protein was removed using ToxinEraserTM Endotoxin Removal Kit (Genescript) following manufacturer's instruction. Protein concentration was determined using BCA Protein Assay Kit (Beyotime, Jiangsu, China).

### Overexpression of MptpB in RAW264.7

HEK293T (4 × 10^6^) was co-transfected with pMSCV-eGFP-MptpB (10 μg) and pCL-Ampho (10 μg) in 10 cm dishes to generate retrovirus carrying MptpB gene. The cell culture supernatant with virus was collected 72 h after transfection and incubated with RAW264.7 cells for 3 h. RAW264.7 cells were then cultured for an additional 72 h, and GFP expressing cells were sorted out by BD FACS AriaTMIII sorter to obtain the RAW264.7-MptpB cell line with more than 98% GFP positive cells. pMSCV-eGFP empty vector plasmid was used in parallel to generate retrovirus without MptpB gene and then RAW264.7-vector cell line expressing GFP but not MptpB.

### Cytokine and NO detection

ELISA kit (Invitrogen) was used to measure mouse IL-1β and IL-6 protein levels in supernatant from RAW264.7 cells unstimulated or stimulated as indicated for 24 h. The level of NO was detected by NO Assay Kit E1030 (Applygen, China) following the manufacturer's instructions.

### Quantitative real-time PCR

TRIzol reagent (Invitrogen) was used to extract RNA from RAW264.7 cells according to the manufacturer's instructions. The mRNA was then reversely transcribed into cDNA with oligo-dT primers (Takara). Real-time PCR was performed using SYBR Premix Ex Taq and LightCycler® 480 to measure IL-6, IL-1β, and IRF1 expression. Primers used in Real-time PCR analysis were as the followings: Mouse IL-6 was amplified with forward primer 5′-TAGTCCTTCCTACCCCAATTTCC-3′ and reverse primer 5′-TTGGTCCTTAGCCACTCCTTC-3′; mouse IL-1β with forward primer 5′-GCAACTGTTCCTGAACTCAACT-3′ and reverse primer 5′-ATCTTTTGGGGTCCGTCAACT-3′; mouse GAPDH with forward primer 5′-GAAGGGCTCATGACCACAGT-3′ and reverse primer 5′-GGATGCAGGGATGATcGTTCT-3′; mouse IRF1 with forward primer 5′-ACCTGGGTCAGGACTTGGAT-3′ and reverse primer 5′-GTTTCCTCGAGGGCTGTCAA-3′. Gene expression was determined by the 2–ΔΔCT method.

### Luciferase assay

RAW264.7 cells were cultured in 6-well plate at 1.3 × 10^5^ cells per well and co-transfected with 0.5 μg pFLAG-cmv2-MptpB plasmid or vector and NF-κB or AP-1 luciferase reporter plasmid (0.45 μg). pRL-TK (500 ng) was used as an internal control. After 24 h, RAW264.7 was stimulated by Pam3CSK4 (10 μg/ml), LPS (100 ng/ml) or H37Rv (MOI = 10) for 12 h. Dual-Luciferase®Reporter Assay System (Promega) was used to measure luminescence from NF-κB-Luc or AP-1-Luc, and the results were normalized by Renilla luminescence following the manufacturer's instruction.

### Western blot analysis

10 or 12% SDS-PAGE were used to separate RAW264.7 lysates and proteins were transferred to polyvinylidene fluoride membrane. p65, p-p65, IKKα, p-IKKα, p53, Erk1/2, p-Erk1/2, p38, p-p38, Caspase3, iNOS, GAPDH, and tubulin antibodies were purchased from Cell Signaling Technology. FLAG antibody was from Sigma. MptpB antibody was produced in our lab. The secondary antibodies were HRP-conjugated anti-mouse or anti-rabbit IgG (Southern-Biotech).

### Caspase activity assay

RAW264.7-MptpB and RAW264.7-vector cells (4 × 10^5^) were treated with IFN-γ (10 ng / ml) for 24 h. Caspase-3 activity was detected using Ac-DEVD-pNA as a substrate (Sigma) according to the manufacturer's protocol.

### Phosphatase activity

The phosphatase activity of mPTPB was determined using pNPP as a substrate at 25°C. Bacterial expressed His-MptpB or Raw264.7 cell expressed FLAG-MptpB immunoprecipitated from RAW264.7-MptpB cell lysate using anti-FLAG beads (Sigma) were added into 200 μl of reaction mixture containing 5 mM pNPP/50 mM sodium acetate (pH5.5)/150 mM NaCl. Reactions were carried out for indicated time and quenched by addition of 50 μl 5N NaOH. The amount of p-nitrophenol product was determined by the absorbance at 405 nm.

### Flow cytometry

RAW264.7-MptpB cells stimulated by BCG (MOI = 10) for 24 h were stained with 7AAD-PerCP-Cy5.5 and Annexin V-PE (Biolegend). The FACS Canto II flow cytometer of FACS Diva software was used to analyze the stained cells.

### Statistical analysis

Data were assessed by GraphPad Prism 5 software from three independent experiments shown as mean ± SD. The two-tailed unpaired *t*-test was used to assess differences between groups. *P*-value < 0.05 means statistically significant. The protein-protein interaction network was established by the STRING database version 10.5 (https://string-db.org). GO over-representative enrichment analysis was performed by PANTHER (Protein Analysis via Evolutionary Relationships).

## Results

### MptpB functioned intracellularly and promoted survival of *Mycobacterium tuberculosis* in the macrophage

**Figure 1:**
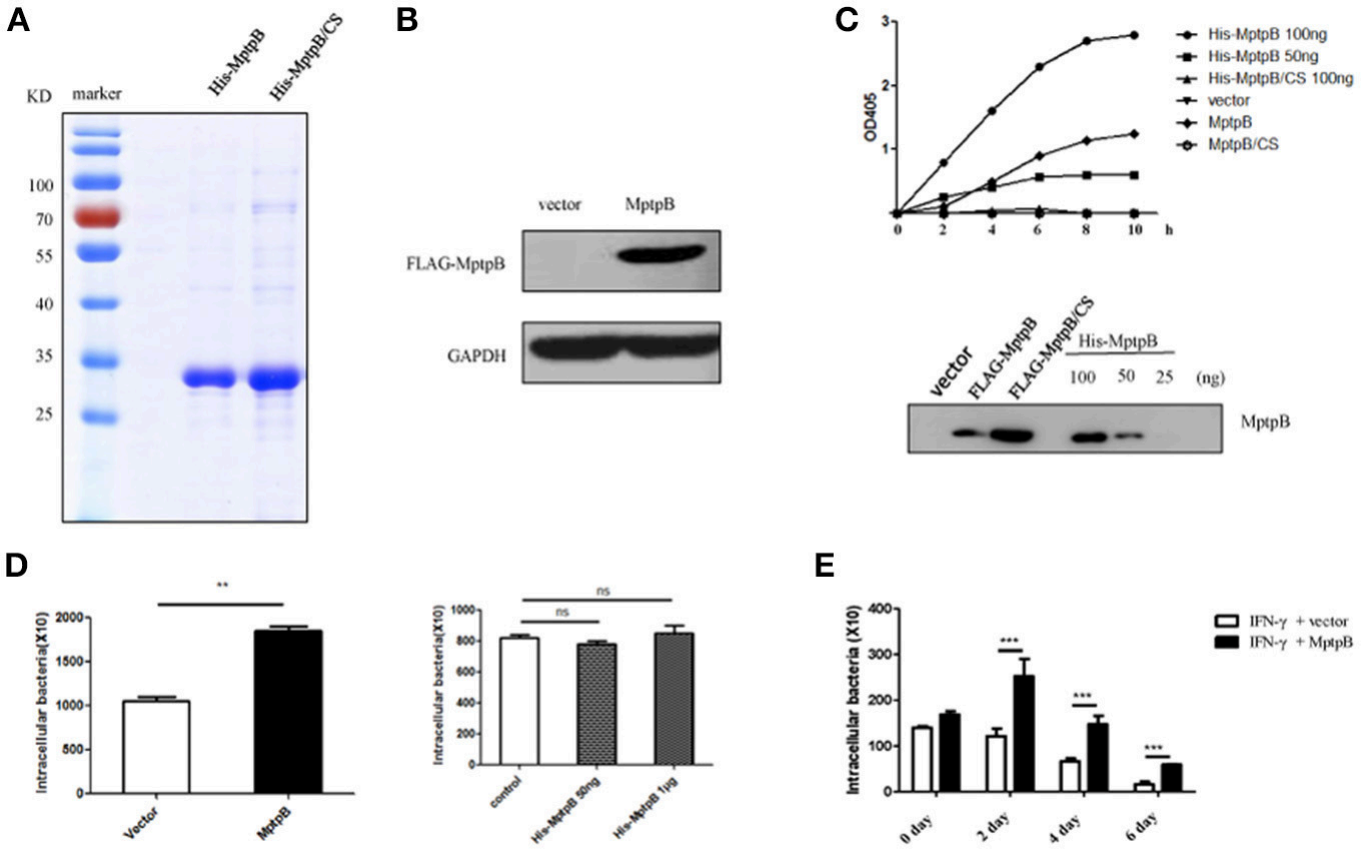
MptpB functioned intracellularly and promoted mycobacterium tuberculosis H37Rv survival in the macrophage. **(A)** Coomassie Blue staining of purified His-MptpB and His-MptpB/CS protein expressed in *E. coli*. **(B)** RAW264.7 cell line stably overexpressed MptpB. **(C)** Phosphatase activity of MptpB expressed by *E. coli* and RAW264.7. *E. coli* expressed His-MptpB and catalytic inactive mutant protein His-MptpB/CS were added into assay system at indicated amount. RAW264.7 cell expressed FLAG-MptpB and FLAG-MptpB/CS were immunoprecipitated from cell lysate using anti-FLAG beads. Assay was carried for indicated time, and the amounts of the product were determined by absorbance at 405 nm. Western blot showed the amount of MptpB protein in assay samples using anti-MptpB antibody. **(D)** IFN-γ activated RAW264.7-MptpB and RAW264.7-vector cells were infected with *M. smegmatis* at MOI = 20. Intracellular bacteria were collected 12 h after infection and CFU was detected by plating on MB7H10 plates. IFN-γ activated RAW264.7 cells were untreated or pretreated with bacteria expressed His-MptpB at indicated dosages for 2 h. Cells were infected with *M. smegmatis* at MOI = 20. Intracellular bacteria were collected 12 h after infection and CFU was detected by plating on MB7H10 plates. **(E)** IFN-γ activated RAW264.7-MptpB and RAW264.7-vector were infected with H37Rv (MOI = 10). At different time points (0, 2, 4, or 6 days), the macrophage was lysed and the CFUs of intracellular Mtb was detected by plating on MB7H10 plates. Data shown in **(D,E)** are mean ± SD of three independent experiments. ^**^P < 0.01; ^***^P < 0.001; ns, not significant.

It has been shown that MptpB is a secreted virulence factor of Mtb. To determine whether MptpB works extracellularly or intracellularly, we decided to purify MptpB expressed by *E. coli* and add it to the culture medium of macrophage, or directly express MptpB inside macrophage, then evaluated their effects on mycobacteria survival. To do this, we first amplified MptpB gene from the genomic DNA of *M. tuberculosis* H37Rv and inserted into bacterial expression vector pET28b with a His-tag at N-terminal. A catalytically inactive C106S mutant of MptpB (MptpB/CS) expression vector was also constructed. Wild type and mutant MptpB proteins were expressed by Rosetta and purified using Ni-NTA beads (Figure 1A). MptpB gene was also inserted into retroviral vector pMSCV-eGFP downstream of the FLAG-tag to construct the pMSCV-eGFP-MptpB plasmid. Retrovirus containing MptpB gene was then produced and used to infect the murine macrophage RAW264.7 cells to generate RAW264.7-MptpB cell line. Expression of MptpB in the cell line was confirmed by western blot using anti-Flag antibody (Figure 1B).

To verify whether MptpB, a bacterial protein, folds correctly when expressed in mammalian cells, we tested the phosphatase activity of MptpB expressed by RAW264.7 cells. Lysates of RAW264.7-vector, RAW264.7-MptpB, and RAW264.7-MptpB/CS cells were subject to immunoprecipitation using FLAG beads. The IP products (FLAG-MptpB) as well as bacterial expressed His-MptpB proteins were then tested for their phosphatase activity using pNPP as the substrate. The results indicated that the IP product from RAW264.7-MptpB showed a significant phosphatase activity, while those from RAW264.7-vector and RAW264.7-MptpB/CS had no phosphatase activity (Figure 1C). The MptpB proteins used in phosphatase assays were then analyzed by western blot using antibody against MptpB (Figure 1C). The amount of FLAG-MptpB protein was about 70 ng (estimated from western blot signals), and its phosphatase assay product curve sit between those from 50 ng and 100 ng of bacterial expressed His-MptpB (Figure 1C), indicating that cell expressed MptpB had a comparable phosphatase activity as bacterial expressed MptpB, suggesting that cell expressed MptpB folded and functioned correctly.

To investigate whether MptpB functions extracellularly or intracellularly, we used *M. smegmatis*, a kind of fast-growing mycobacteria, to infect IFN-γ activated RAW264.7-vector and RAW264.7-MptpB cells, as well as RAW264.7 cells untreated or treated with bacteria expressed MptpB. The amount of endogenous MptpB in 1 × 10^6^ H37Rv (used to infect 1 × 10^5^ cells at MOI = 10) is about 50 ng (Figure S1), we therefore treated 1 × 10^5^ cells with a lower dosage of 50 ng and a higher dosage of 1 μg of bacterial expressed His-MptpB. The results showed that extracellularly applied MptpB, at both dosages, had no effect on M. smegmitis survival, while intracellular expressed MptpB significantly increased the bacterial load inside the macrophage (Figure 1D), indicating that MptpB functions inside the cells.

To determine whether intracellular MptpB also promotes the survival of virulent strain H37Rv, we assessed the bacterial loads in IFN-γ activated RAW264.7-vector and RAW264.7-MptpB cells 2, 4, and 6 days after H37Rv infection (MOI = 10). Results showed that the CFU of bacteria in RAW264.7-MptpB cells was significantly higher compared to the control group (Figure 1E, ^***^p < 0.001) indicating that MptpB can promote the survival of H37Rv in activated macrophage. We also tested the intracellular bacteria burdens of H37Rv in RAW264.7-MptpB without IFN-γ treatment, and found no difference when compared to the control group (Figure S2), indicating that MptpB played an important role in survival of H37Rv in activated macrophages.

### Signal pathways potentially regulated by MptpB

**Figure 2:**
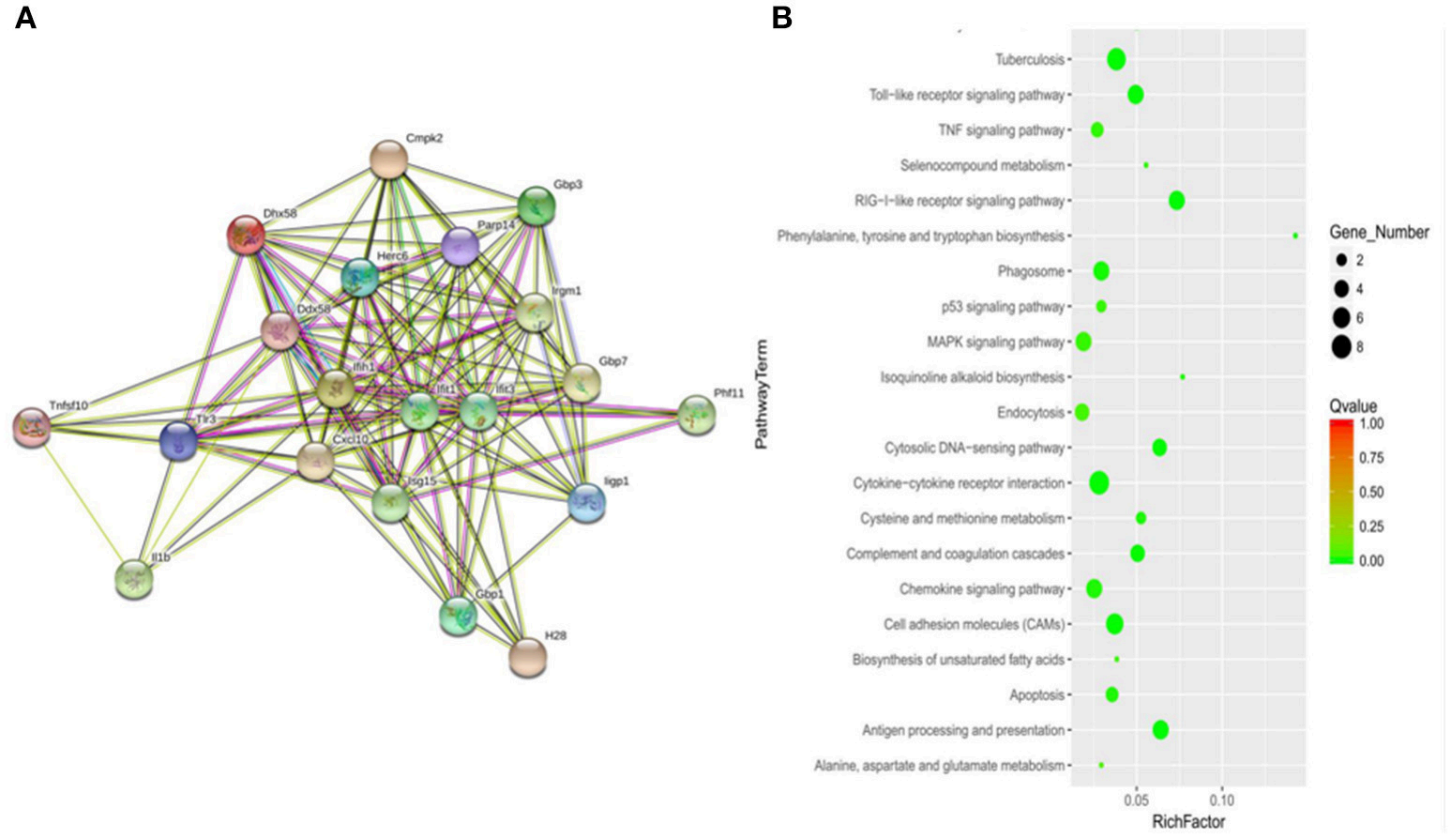
Signal pathways potentially regulated by MptpB. **(A)** Network analysis of genes potentially regulated by MptpB. A protein–protein interaction network analysis for differentially expressed genes in H37Rv infected RAW264.7-Vector and RAW264.7-MptpB cells were performed using STRING 10.0 based on evidence channels **(B)** The signaling pathways enriched by MptpB regulated genes were identified by Gene Ontology (GO) enrichment analysis.

To determine how MptpB affects the host immune responses, we focused our attention on the signal pathways and proteins potentially regulated by MptpB. RAW264.7-vector and RAW264.7-MptpB cells were activated by IFN-γ for 16 h, stimulated by H37Rv (MOI = 10) for 6 h, and subject to RNA extraction. Expression levels of all protein coding genes in the two cell lines were determined by Next Generation Sequencing (NGS), and 75 differentially expressed genes were identified. These genes are potentially regulated by MptpB. To investigate how MptpB might regulate the expression of these genes, we conducted a protein–protein interaction network analysis for the target genes using STRING 10.0, a bioinformatic tool that maps protein–protein associations based on evidence channels (Figure 2A). Gene Ontology (GO) enrichment analysis (Harris et al., 2004) was also performed to classify the functions of those 75 proteins. The results showed that a large number of target proteins (such as IL-1β, Arrb1, and TLR3) are regulated by pattern recognition receptor (PRR) signal pathways (Figure 2B). In addition, some proteins are associated with cell apoptosis (such as TNFRSF10; Figure 2B). These results suggested that MptpB might realize its function by affecting inflammation response induced by PRR and apoptosis of host cells.

### MptpB inhibited the expression of inflammatory cytokines and iNOS

**Figure 3:**
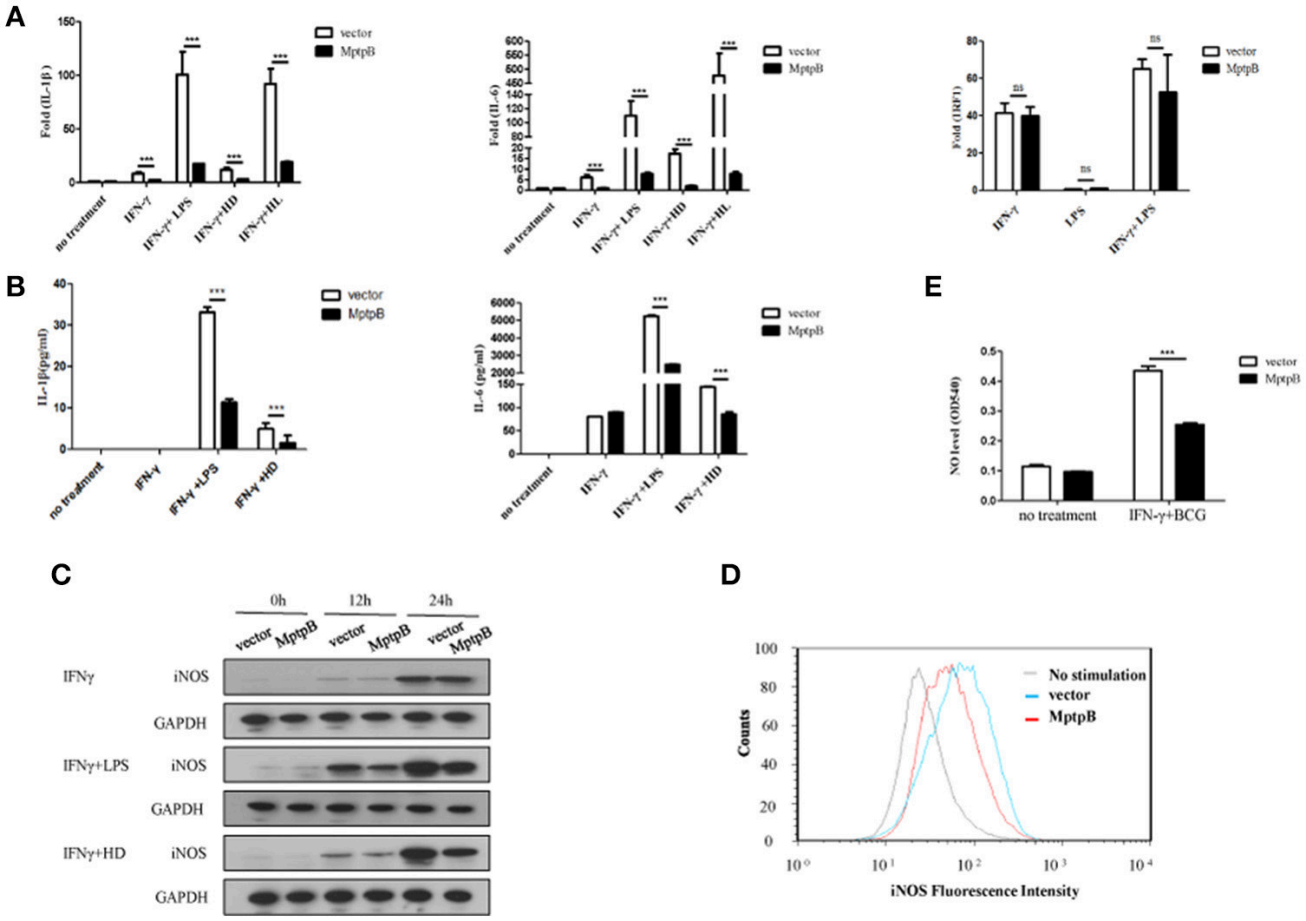
MptpB inhibited the expression of inflammatory cytokines IL-1β, IL-6 and iNOS **(A)** Real-time PCR analysis of IL-1β, IL-6, and IRF1 mRNA expression in RAW264.7-Vector or RAW264.7-MptpB cells stimulated with IFN-γ, IFN-γ + LPS, IFN-γ + HD (heat inactivated H37Rv), or IFN-γ + HL (H37Rv) for 6 h. **(B)** ELISA analysis of IL-1β and IL-6 protein amounts in culture media from RAW264.7-Vector or RAW264.7-MptpB cells stimulated with IFN-γ, IFN-γ + LPS, or IFN-γ + HD (heat inactivated H37Rv) for 24 h. **(C)** Western blot analysis of iNOS protein levels in RAW264.7-Vector or RAW264.7-MptpB cells stimulated with IFN-γ, IFN-γ +LPS, or IFN-γ + HD for indicated time. **(D)** Flow cytometry analysis of iNOS protein amounts in RAW264.7-Vector or RAW264.7-MptpB cells infected with BCG for 24 h or not. **(E)** Detection of the levels of NO in culture media from RAW264.7-Vector or RAW264.7-MptpB cells stimulated with IFN-γ + BCG for 24 h or not. Data shown in **(A,B,E)** are mean ± SD of three independent experiments. ^***^P < 0.001; ns, not significant.

Mtb infection activates multiple PRRs (majorly TLR2 and TLR4) which lead to induction of inflammatory cytokines and iNOS expression in macrophage. In order to determine whether MptpB affects the PRR signal pathways, we first checked the expression of inflammatory cytokines and iNOS in the macrophage. We treated the RAW264.7-MptpB and RAW264.7-Vector with IFN-γ only, IFN-γ + LPS, IFN-γ + heat inactivated H37Rv (to eliminate endogenous MptpB) (IFN-γ + HD), or IFN-γ + H37Rv (MOI = 10) (IFN-γ + HL), respectively, for 6 h. RNAs were extracted from each sample and gene expression levels were determined by realtime PCR. The results revealed that mRNA expressions of IL-1β and IL-6 in RAW264.7-MptpB cells were significantly lower than those in RAW264.7-Vector cells (Figure 3A, ^***^p < 0.001). IL-1β and IL-6 protein levels were also determined by ELISA after 24 h stimulation of the cells, showing a significantly decreased cytokine amount in RAW264.7-MptpB cells, consistent with the results of mRNA (Figure 3B, ^***^p < 0.001). The expression of a typical IFN-γ inducible gene IRF1, however, had no difference between the two cell lines (Figure 3A). These results suggested that MptpB inhibited LPS or H37Rv induced IL-1β and IL-6 expression in IFN-γ-activated macrophages at the transcriptional level, without affecting the expression of IFN-γ inducible genes.

Mtb exists in phagosome in macrophages. Under the excitation of phagocytosis, cell breathing outbreak occurs and produces reactive nitrogen intermediates, which have severe cytotoxic effect to kill Mtb. The generation of reactive nitrogen intermediates relies on iNOS, whose expression is inducible by both TLR and IFN-γ. In order to further determine the function of MptpB, we detected the expression of iNOS in RAW264.7-MptpB cells. The result showed the expression of iNOS in RAW264.7-MptpB was significantly lower than that in RAW264.7-Vector with IFN-γ+LPS or IFN-γ+HD treatment but not IFN-γ alone (Figure 3C). Moreover, by FACS analysis, we found that the iNOS expression induced by BCG in activated macrophages overexpressing MptpB was reduced compared to the control group (Figure 3D). The amount of NO in the cell culture supernatant of activated RAW264.7-MptpB was also much lower than that in RAW264.7-Vector after BCG stimulation (Figure 3E, ^***^p < 0.001), consistent with the results of iNOS expression. These results revealed that MptpB reduced the expression of iNOS induced by TLR agonist or BCG in activated macrophages and inhibited the generation of destroying reactive nitrogen intermediate NO, thus promoted the survival of Mtb.

### MptpB hampered the NF-κB and MAPK signal pathways

IFN-γ is the predominant inflammatory cytokine contributing to macrophages antimicrobial activity against diverse intracellular pathogens. Given the observation that Mtb survived better in IFN-γ activated macrophages with overexpressed MptpB, there exists a possibility that MptpB might affect IFN-γ signal pathway. To test this, we stimulated RAW264.7-MptpB and its vector control with IFN-γ and looked at the phosphorylation levels of STAT1. Treatment of macrophages with IFN-γ resulted in significant activation of STAT1. Its phosphorylation levels, however, had no difference between the two cell lines for the indicated time periods (Figure S3A), which was consistent with the previous study (Zhou et al., 2010). Together with the above result that the expression of IFN-γ inducible gene IRF1 showed no significant difference between RAW264.7-MptpB and RAW264.7-Vector cells (Figure 3A), we believe that MptpB does not interfere with the IFN-γ signal pathway.

**Figure 4:**
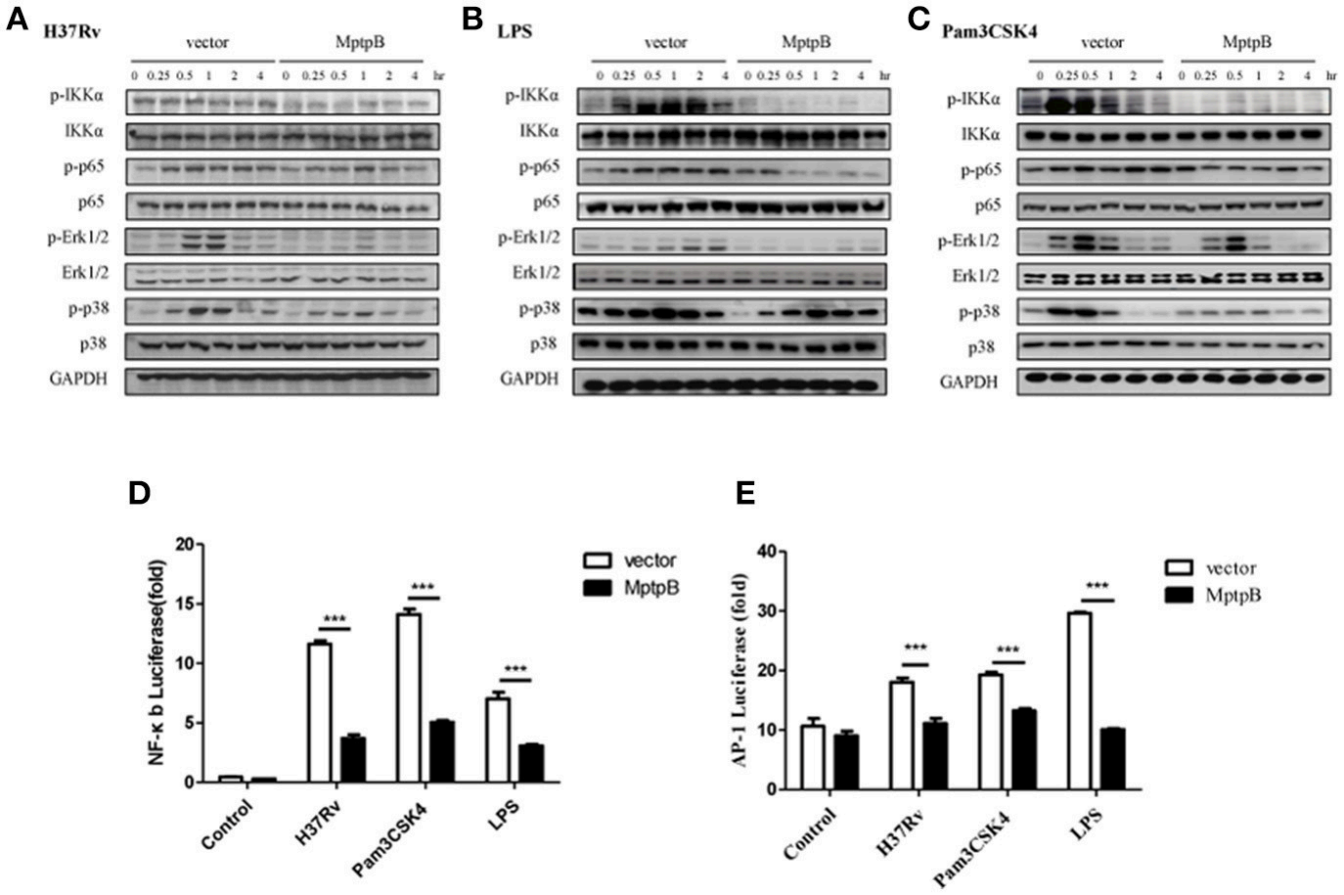
MptpB hampered the NF-κB and MAPK signal pathways. **(A–C)** RAW264.7-Vector or RAW264.7-MptpB cells were stimulated with H37Rv (MOI = 10) **(A)**, LPS **(B)**, or Pam3CSK4 **(C)** for the indicated time periods (0, 0.25, 0.5, 1, 2, or 4 h). The protein levels of p-IKKα, IKKα, p-p65, p65, p-Erk1/2, Erk1/2, p-p38, and p38 in the two cell lines were determined by western blot. **(D–E)** RAW264.7 cells were transfected with the MptpB expression plasmid or vector and internal control Renilla luciferase reporter, plus NF-κB or AP-1 luciferase reporters, respectively. Twenty four hours after transfection, the cells were untreated or treated with H37Rv (MOI = 10), Pam3CSK4 or LPS for 12 h. Luminescence from NF-κB or AP-1 luciferase and Renilla luciferase were measured, and NF-κB **(D)** or AP-1 **(E)** Relative Luciferase Activity were calculated. Data shown in **(D,E)** are mean ± SD of three independent experiments. ^***^P < 0.001.

Mtb induces the production of IL-6 and IL-1β by activating various pattern recognition receptors, especially TLR in macrophages (Harding and Boom, 2010). The pattern recognition receptor activates the NF-κB and MAPK signal pathways, causing the production of IL-1β and IL-6 (Cai et al., 2015). The expression of iNOS can also be induced by the NF-κB signal pathway. Therefore, we analyzed the activation of NF-κB and MAPK signal pathways in RAW264.7-MptpB after H37Rv infection. IFN-γ activated RAW264.7-MptpB and RAW264.7-Vector cells were stimulated with H37Rv (MOI = 10), and the activation status of signaling molecules in NF-κB and MAPK pathways were determined by western blot. The results indicated that MptpB inhibited p65, Erk1/2 and p38 phosphorylation in macrophages upon H37Rv infection (Figure 4A).

Multiple pattern recognition receptors (PRRs) in macrophages activate NF-κB and MAPK signal pathways. As H37Rv majorly activates TLR2 and TLR4, we analyzed the activation of these two TLR signal pathways in cells with or without MptpB expression. We treated RAW264.7-MptpB and RAW264.7-Vector cells with TLR2 agonists Pam3CSK4 or TLR4 agonists LPS, respectively, and checked NF-κB and MAPK signal pathway activation. The results showed that both TLR2 and TLR4 mediated phosphorylation of IKKα, p65, Erk1/2 and p38 were inhibited in RAW264.7-MptpB cells (Figures 4B,C), indicating that MptpB inhibited both TLR2 and TLR4 induced NF-κB and MAPK signal pathway activation. Interestingly, phosphorylation of JNK, the other MAPK activated by TLR, was not significantly impacted by MptpB for the indicated time periods (Figure S3B).

To determine whether the attenuated signal molecule phosphorylation affects target gene expression, we tested the luciferase activities of NF-κB-Luc and AP-1-Luc, respectively, in RAW264.7-MptpB and RAW264.7-Vector stimulated with H37Rv, Pam3CSK4 or LPS. As shown in Figures 4D,E, RAW264.7-MptpB showed lower luciferase activities from both NF-κB-Luc and AP-1-Luc constructs with H37Rv, Pam3CSK4, or LPS stimulation compared with the control cells, indicating that H37Rv and TLR induced NF-κB and AP-1 transactivations were significantly reduced with MptpB expression (Figures 4D,E). The above results revealed that the production of inflammatory cytokines and iNOS in H37Rv infected RAW264.7 cells was inhibited by MptpB via suppressing TLR induced NF-κB and MAPK signal pathway activation.

### MptpB inhibited the apoptosis of the macrophage

**Figure 5:**
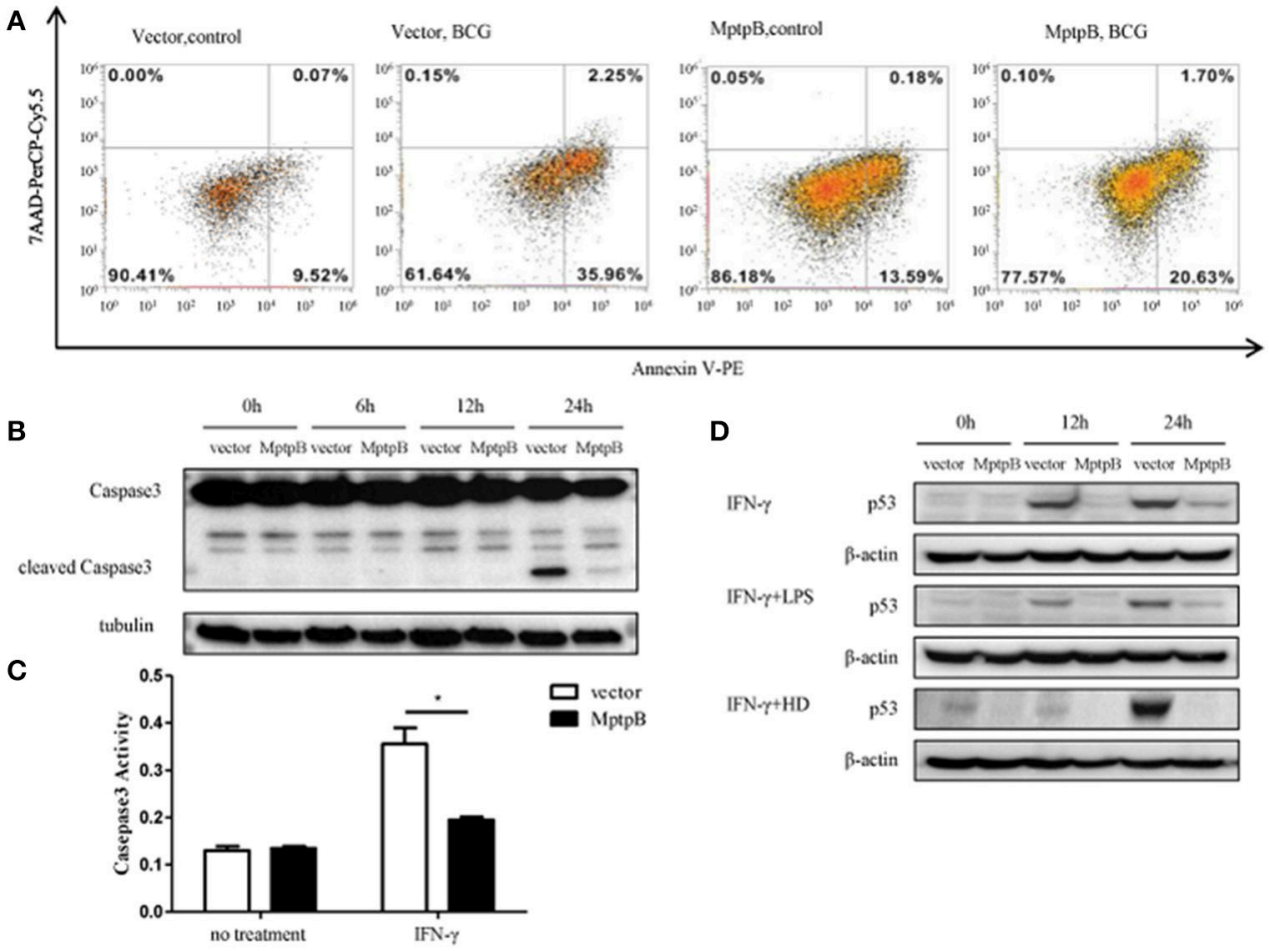
MptpB inhibited the apoptosis of macrophages. **(A)** RAW264.7-Vector and RAW264.7-MptpB were infected with BCG for 24 h before stained with Annexin V-PE/7-AAD. Cells were then subject to Flow cytometry analyses for apoptotic cells. **(B)** RAW264.7-Vector and RAW264.7-MptpB cells were stimulated with BCG for indicated time. The levels of Caspase 3 and cleaved Caspase 3 were determined by western blot. **(C)** RAW264.7-Vector and RAW264.7-MptpB cells were untreated or stimulated by IFN-γ (10 n g/ml) for 24 h. Caspase 3 activities in two cell lines were then detected by Caspase 3 activity kit. **(D)** RAW264.7-Vector and RAW264.7-MptpB were stimulated with IFN-γ, IFN-γ + LPS, or IFN-γ + HD (heat inactivated H37Rv) or not for indicated time. The levels of p53 were determined by western blot. Data shown in **(C)** are mean ± SD of three independent experiments. ^*^P < 0.05.

As in the network analysis apoptosis is also one of the signal pathways potentially regulated by MptpB, we investigated whether MptpB can affect Mtb induced apoptosis in macrophages. We infected RAW264.7-MptpB and RAW264.7-Vector cells with BCG for 24 h, and apoptotic cells were identified by Annexin V /7-AAD staining followed by FACS analysis. We found that the percentage of apoptotic cells in RAW264.7-MptpB was much lower than that in RAW264.7-Vector (22.33 vs. 38.21%) (Figure 5A), suggesting that MptpB decreased BCG induced apoptosis in macrophages. To provide further evidence that MptpB inhibits the apoptosis of macrophages, we checked the cleaved Caspase3 protein in RAW264.7-Vector and RAW264.7-MptpB cells treated with IFN-γ and BCG by western blot (Figure 5B). The results showed that the protein level of cleaved Caspase 3 was much lower in RAW264.7-MptpB cells, indicating that MptpB inhibited the activation of Caspase3. This is confirmed by Caspase3 activity assay, which indicated that the activity of Caspase 3 is lower in RAW264.7-MptpB cells (Figure 5C, ^*^p < 0.05), consistent with the FACS and western blot results.

As p53 plays a critical role in regulating apoptosis and appears to be potentially regulated by MptpB (Figure 2B), we analyzed the p53 protein levels in differently stimulated RAW264.7-MptpB and RAW264.7-Vector cells. We found that MptpB decreased p53 protein levels induced by either IFN-γ or IFN-γ + LPS/HD (Figure 5D), suggesting MptpB suppressed macrophage apoptosis at least partially by reducing the expression of p53, therefore promoted the survival of the *M. tuberculosis*.

## Discussion

Macrophages are primary innate immune cells that contribute to the host immunity against mycobacterial infections by recognizing, engulfing, and eventually killing invasive Mtb (Hmama et al., 2015; Shen et al., 2016; Liu et al., 2017). Recent studies have shown that macrophage is a main sanctuary to mycobacteria as it establishes various mechanisms to respond to the invasion of pathogens as well as regulate host fate (Killick et al., 2013; Dey and Bishai, 2014; Xu et al., 2014; Arbués et al., 2016; Liu et al., 2017). The interaction between mycobacteria and macrophages determines the progression of infection.

MptpB, one of the PTP family members, is a negative modulator of macrophages in innate immunity (Zhou et al., 2010). In accordance with the previous research (Singh et al., 2003), we found MptpB serves as a virulence factor contributing to the survival of Mtb in activated macrophages (Figure 1E). Macrophages can produce a series of inflammatory factors under Mtb infection. Growing evidence indicates that IL-6 and IL-1β secreted by macrophages are critical to produce an adaptive response to mycobacteria, eventually resulting in host resistance (Juffermans et al., 2000; Flynn and Chan, 2001; North and Jung, 2004; Fremond et al., 2007; Cooper, 2009; Wang et al., 2015). We demonstrated that MptpB inhibited the expression of proinflammatory cytokines IL-1β and IL-6 in activated RAW264.7 cells (Figures 3A,B), and the curtailed production of proinflammatory cytokines as well as iNOS promoted the survival of Mtb in macrophage.

Macrophages can recognize multiple exogenous pathogens by PRRs. TLR2 and TLR4 have been reported to be involved in the recognition of Mtb (Chen et al., 2012). TAK1 downstream of TLR activates NF-κB and MAPK signaling pathways by phosphorylating IKK and MAPK, thereby induces the expression of inflammatory factors (Wang et al., 2001; Xi et al., 2010; Chen et al., 2017; Peng et al., 2017). It has been reported that MptpB can block the phosphorylation of p38 and Erk in macrophages infected with Mtb(Zhou et al., 2010). Our data revealed that MptpB can suppress the activation of both NF-κB and MAPK signal pathways induced by TLR-2/TLR-4 agonists or Mtb, therefore hampered the anti-bacterial functions of macrophages (Figures 4A,B). In addition to inflammatory cytokines, we also found that Gng12, Rps6ka2, and Arrb1 were significantly modulated by MptpB at the transcriptional level in H37Rv infected macrophages. These genes are involved in MAPK signal pathways. Gng12 is a negative modulator of LPS induced inflammation in neurodegenerative diseases (Larson et al., 2010). Rps6ka2 has been reported to function downstream of Erk signal pathway and result in decreased apoptosis in pancreatic cancer (Milosevic et al., 2013). Arrb1, a signal adaptor, scaffolds a variety of kinases in MAPK cascade including Erk2, MEK1, and Raf1. It directly binds Erk2 and promotes the activation of Erk1/2 (Coffa et al., 2011). The data implied that MptpB might interfere with MAPK pathway via multiple mechanisms.

It should be mentioned that we haven't found proteins directly interacting with MptpB in macrophages. By co-immunoprecipitation and mass spectrometry, we obtained several candidate proteins that might interact with MptpB, including TAB1, Rab11, DDX3 and DDX5. Further studies needs to be done to identify the interacting protein and substrate of MptpB in macrophage.

In conclusion, our data demonstrated that MptpB expression promoted the survival of the Mtb in activated macrophages. MptpB served as a negative regulator of Mtb-induced inflammation and apoptosis in macrophages via inhibiting NF-κB and MAPK signaling pathways. The decreased inflammatory response and apoptosis resulted from MptpB expression contributed to mycobacteria H37Rv survival. The research provided novel insights into the mechanism of MptpB functions as well as the interaction between mycobacteria and host cells.

## Author contributions

SX and YD designed the study. LF performed the experiments mostly. CJ, XW and FL helped to complete the experiments. LF, SX and YD interpreted the data. LF, SX and YD wrote the manuscript. SX and YD authorized the final version of this paper.

### Conflict of interest statement

The authors declare that the research was conducted in the absence of any commercial or financial relationships that could be construed as a potential conflict of interest.

## Supplementary material

Figure S1 Western blot analysis of endogenous MptpB protein expressed in 1 × 106 H37Rv. The protein concentration of *E. coli* expressed His-MptpB was determined by BCA kit. Membrane was blotted using anti-MptpB antibody.

Figure S2 Resting RAW264.7-MptpB and RAW264.7-vector were infected with H37Rv (MOI = 10). At different time points (0, 2, 4, or 6 days), the macrophage was lysed and the CFUs of intracellular Mtb was detected by plating on MB7H10 plates. Data shown are mean ± SD of three independent experiments. ns, not significant.

Figure S3 MptpB had no effect on the phosphorylation of STAT1 and JNK in the macrophage. **(A)** The expression of p-STAT1 and STAT1 in RAW264.7-Vector or RAW264.7-MptpB treated by IFN-γ for the indicated time periods was determined by western blot. **(B)** The levels of p-JNK and JNK in RAW264.7-Vector or RAW264.7-MptpB by treated by LPS for the indicated time periods were determined by western blot.
