# Supplementary figures and images for: MptpB Promotes Mycobacteria Survival by Inhibiting the Expression of Inflammatory Mediators and Cell Apoptosis in Macrophages
Source: Front Cell Infect Microbiol. 2018 May 25;8:171. doi: 10.3389/fcimb.2018.00171 (PMC5981270; doi:10.3389/fcimb.2018.00171)

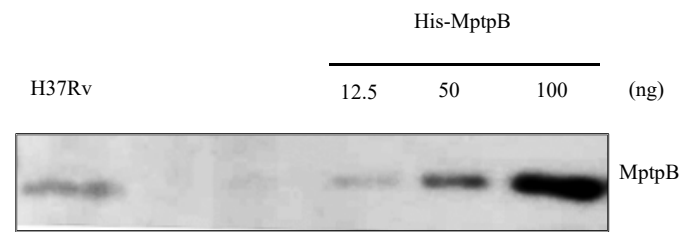

Supplement: Figure S1 — Western blot analysis of endogenous MptpB protein expressed in 1 × 10^6^ H37Rv. The protein concentration of E. coli expressed His-MptpB was determined by BCA kit. Membrane was blotted using anti-MptpB antibody. [file Image_1.PDF]

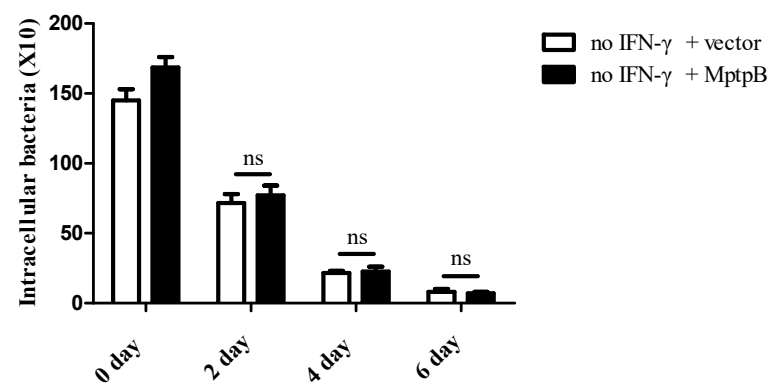

Supplement: Figure S2 — Resting RAW264.7-MptpB and RAW264.7-vector were infected with H37Rv (MOI = 10). At different time points (0, 2, 4, or 6 days), the macrophage was lysed and the CFUs of intracellular Mtb was detected by plating on MB7H10 plates. Data shown are mean ± SD of three independent experiments. ns, not significant. [file Image_2.PDF]

**A**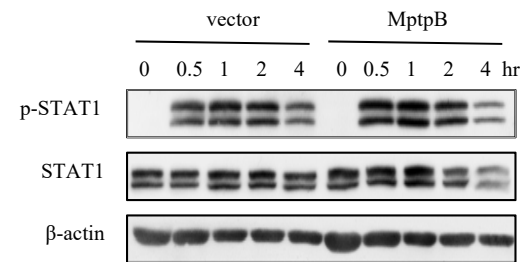**B**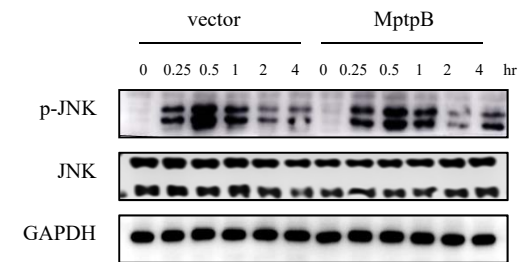

Supplement: Figure S3 — MptpB had no effect on the phosphorylation of STAT1 and JNK in the macrophage. (A) The expression of p-STAT1 and STAT1 in RAW264.7-Vector or RAW264.7-MptpB treated by IFN-γ for the indicated time periods was determined by western blot. (B) The levels of p-JNK and JNK in RAW264.7-Vector or RAW264.7-MptpB by treated by LPS for the indicated time periods were determined by western blot. [file Image_3.PDF]
