# Supplementary material for: MptpB Promotes Mycobacteria Survival by Inhibiting the Expression of Inflammatory Mediators and Cell Apoptosis in Macrophages
Source: Front Cell Infect Microbiol. 2018 May 25;8:171. doi: 10.3389/fcimb.2018.00171 (PMC5981270; doi:10.3389/fcimb.2018.00171)
Supplement: Supplementary file 4 [file Data_Sheet_1.DOCX]

Supplementary Material

**The mycobacterial phosphatase MptpB promotes Mtb survival by inhibiting the expression of inflammatory mediators and cell apoptosis in the macrophage**

**Lingbo Fan, Xiaoyu Wu, Chunyan Jin, Fengge Li, Sidong Xiong^*^, Yuanshu Dong^*^**

*** Correspondence:** Sidong Xiong: sdxiongfd@126.com, Yuanshu Dong: ysdong@suda.edu.cn

# Supplementary Figures

## Supplementary Figures


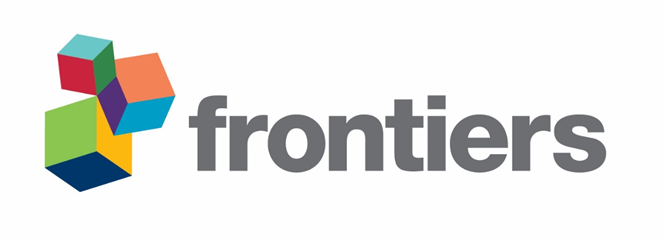


**Supplementary Figure 1.** Western blot analysis of endogenous MptpB protein expressed in 1x10^6^ H37Rv. The protein concentration of E.coli expressed His-MptpB was determined by BCA kit. Membrane was blotted using anti-MptpB antibody.

**Supplementary Figure 2**.Resting RAW264.7-MptpB and RAW264.7-vector were infected with H37Rv (MOI = 10). At different time points (0, 2, 4 or 6 days), the macrophage was lysed and the CFUs of intracellular Mtb was detected by plating on MB7H10 plates. Data shown are mean ± SD of three independent experiments. ns, not significant.

**Supplementary Figure 3.** MptpB had no effect on the phosphorylation of STAT1 and JNK in the macrophage **(A)** The expression of p-STAT1 and STAT1 in RAW264.7-Vector or RAW264.7-MptpB treated by IFN-γ for the indicated time periods was determined by western blot. **(B)** The levels of p-JNK and JNK in RAW264.7-Vector or RAW264.7-MptpB by treated by LPS for the indicated time periods were determined by western blot.
