# Supplementary material for: MptpB Promotes Mycobacteria Survival by Inhibiting the Expression of Inflammatory Mediators and Cell Apoptosis in Macrophages
Source: Front Cell Infect Microbiol. 2018 May 25;8:171. doi: 10.3389/fcimb.2018.00171 (PMC5981270; doi:10.3389/fcimb.2018.00171)
Supplement: Supplementary file 5 [file Presentation_1.PPTX]

## Slide 1
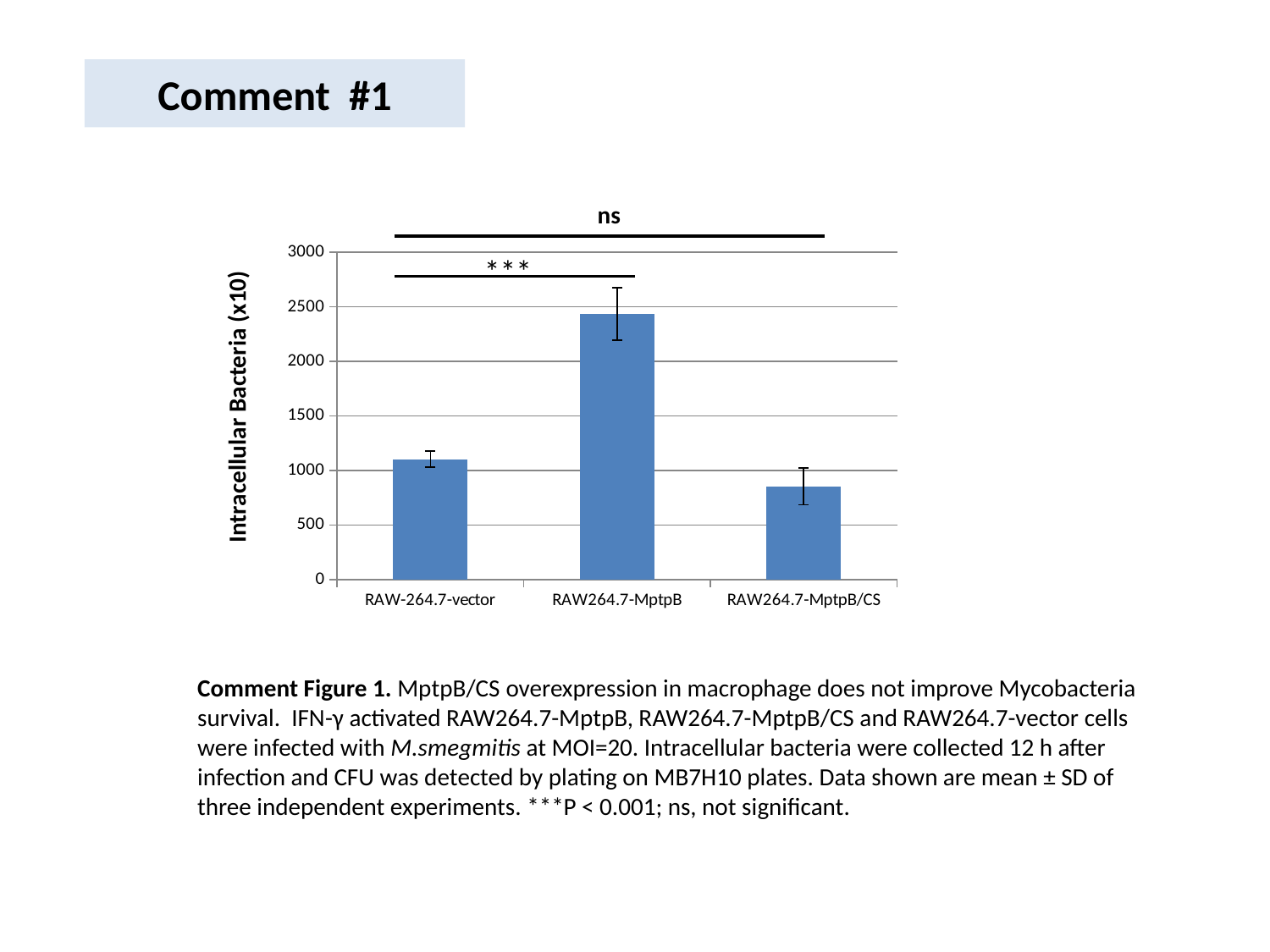

Comment #1
ns
### Chart
| Category | |
|---|---|
| RAW-264.7-vector | 1103.3333333333333 |
| RAW264.7-MptpB | 2436.6666666666665 |
| RAW264.7-MptpB/CS | 853.3333333333334 |***
Intracellular Bacteria (x10)
Comment Figure 1. MptpB/CS overexpression in macrophage does not improve Mycobacteria survival. IFN-γ activated RAW264.7-MptpB, RAW264.7-MptpB/CS and RAW264.7-vector cells were infected with M.smegmitis at MOI=20. Intracellular bacteria were collected 12 h after infection and CFU was detected by plating on MB7H10 plates. Data shown are mean ± SD of three independent experiments. ***P < 0.001; ns, not significant.

## Slide 2
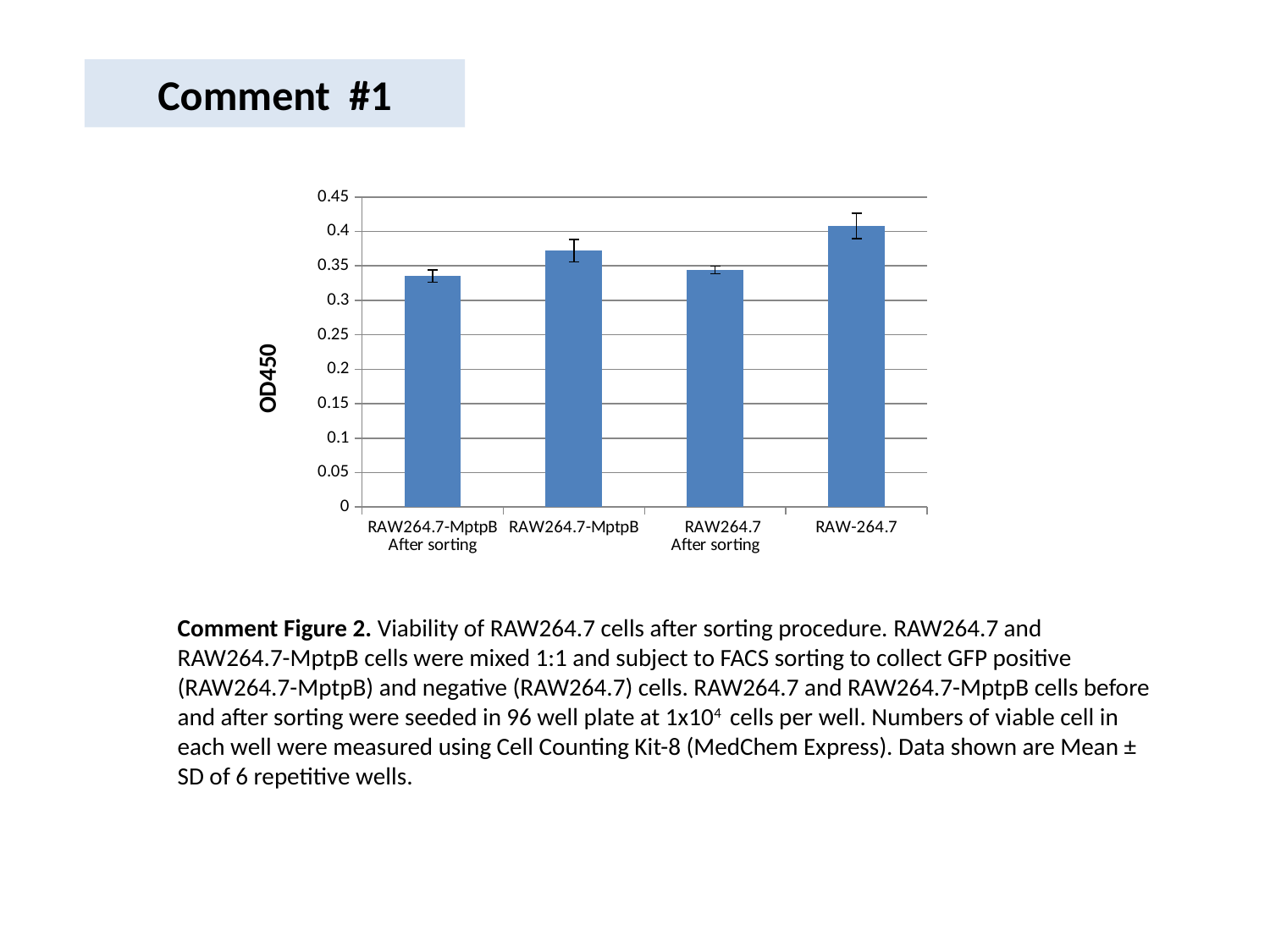

Comment #1
### Chart
| Category | |
|---|---|
| RAW264.7-MptpB After sorting | 0.33525000000000005 |
| RAW264.7-MptpB | 0.372 |
| RAW264.7 After sorting | 0.34425000000000006 |
| RAW-264.7 | 0.40825 |OD450
Comment Figure 2. Viability of RAW264.7 cells after sorting procedure. RAW264.7 and RAW264.7-MptpB cells were mixed 1:1 and subject to FACS sorting to collect GFP positive (RAW264.7-MptpB) and negative (RAW264.7) cells. RAW264.7 and RAW264.7-MptpB cells before and after sorting were seeded in 96 well plate at 1x10^4^ cells per well. Numbers of viable cell in each well were measured using Cell Counting Kit-8 (MedChem Express). Data shown are Mean ± SD of 6 repetitive wells.

## Slide 3
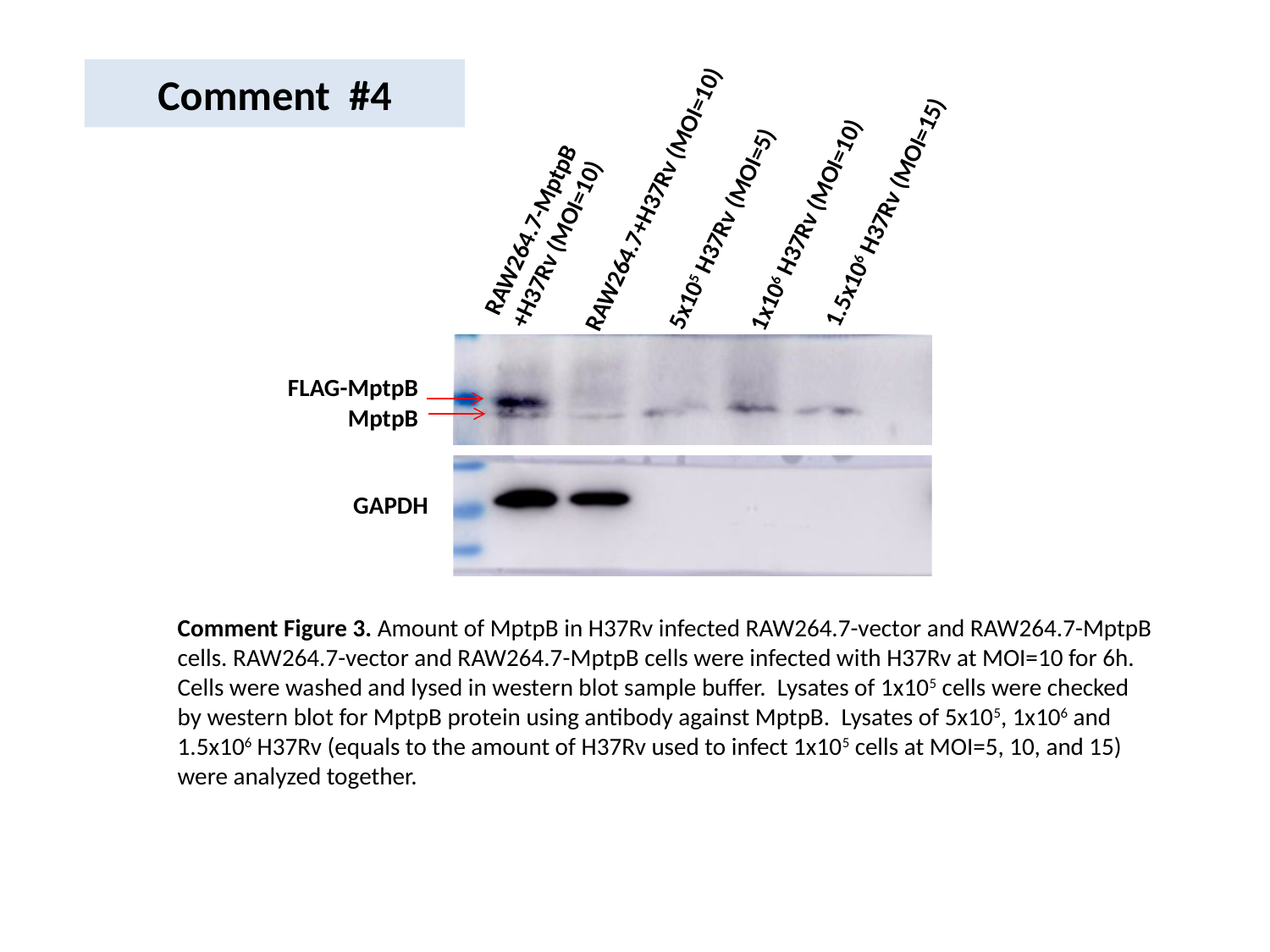

Comment #4
RAW264.7+H37Rv (MOI=10)
1.5x10^6^ H37Rv (MOI=15)
RAW264.7-MptpB
+H37Rv (MOI=10)
1x10^6^ H37Rv (MOI=10)
5x10^5^ H37Rv (MOI=5)
FLAG-MptpB
MptpB
GAPDH
Comment Figure 3. Amount of MptpB in H37Rv infected RAW264.7-vector and RAW264.7-MptpB cells. RAW264.7-vector and RAW264.7-MptpB cells were infected with H37Rv at MOI=10 for 6h. Cells were washed and lysed in western blot sample buffer. Lysates of 1x10^5^ cells were checked by western blot for MptpB protein using antibody against MptpB. Lysates of 5x10^5^, 1x10^6^ and 1.5x10^6^ H37Rv (equals to the amount of H37Rv used to infect 1x10^5^ cells at MOI=5, 10, and 15) were analyzed together.

## Slide 4
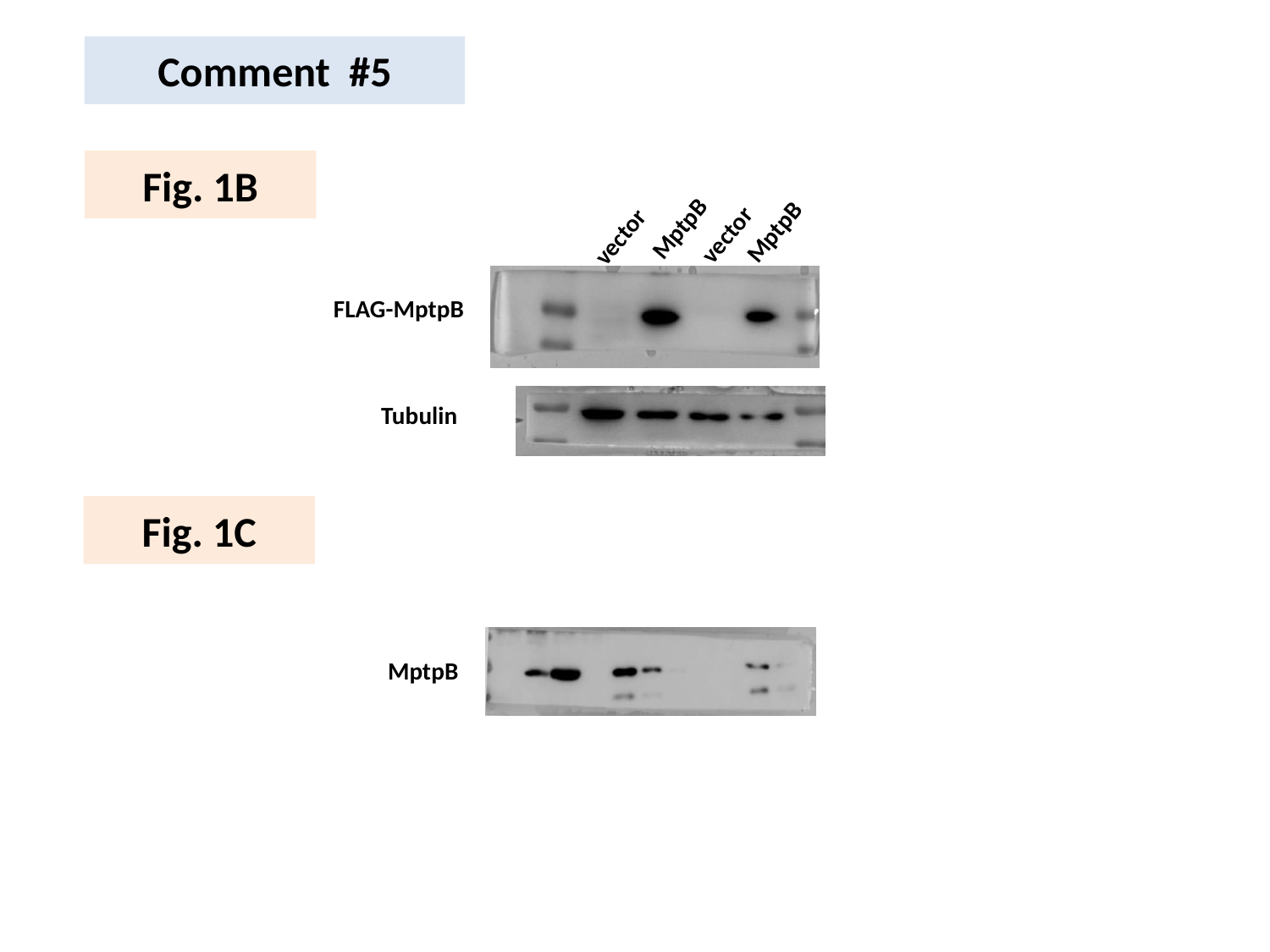

Comment #5
Fig. 1B
MptpB
MptpB
vector
vector
FLAG-MptpB
Tubulin
Fig. 1C
MptpB

## Slide 5
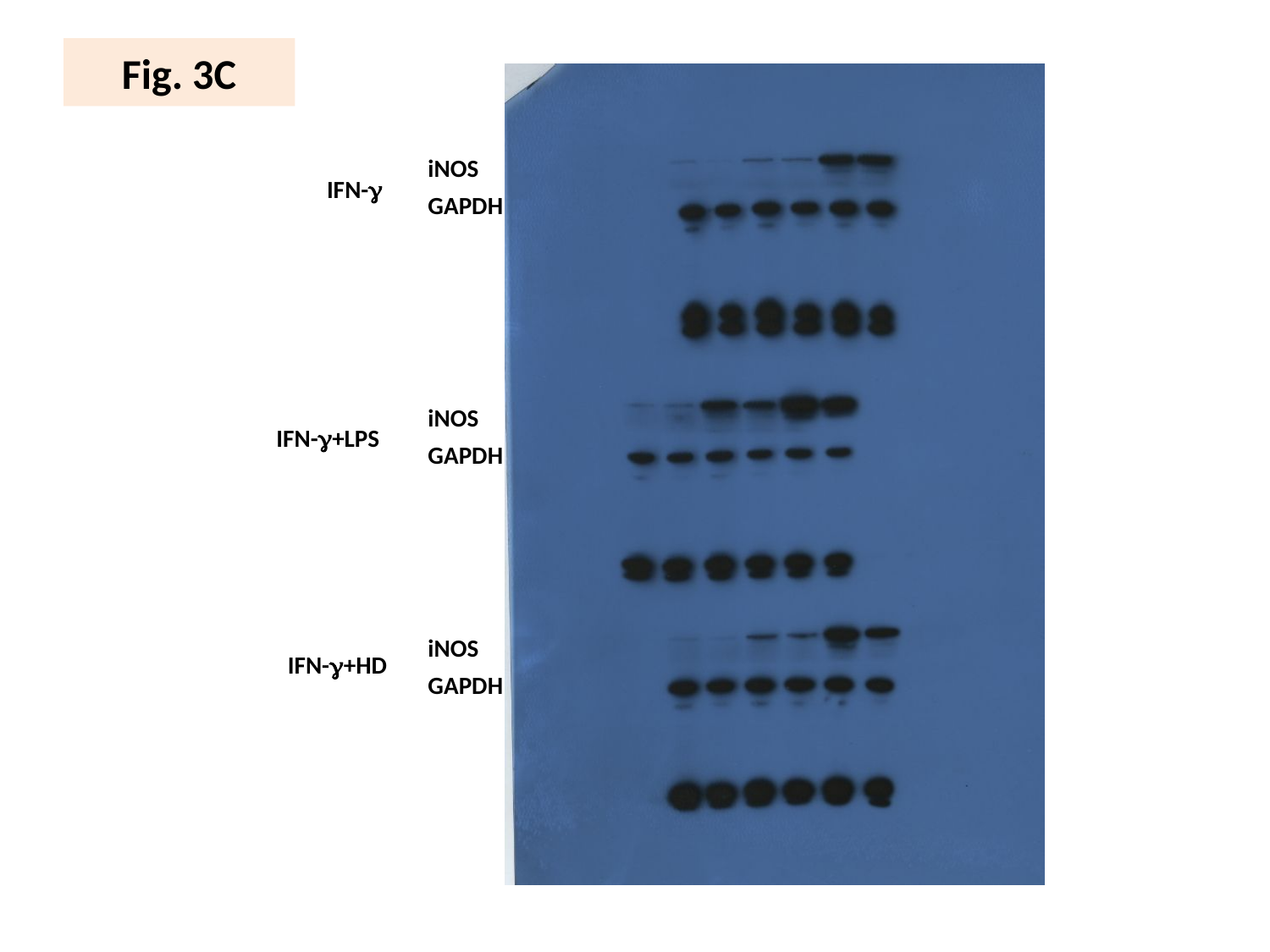

# Fig. 3C
iNOS
IFN-g
GAPDH
iNOS
IFN-g+LPS
GAPDH
iNOS
IFN-g+HD
GAPDH

## Slide 6
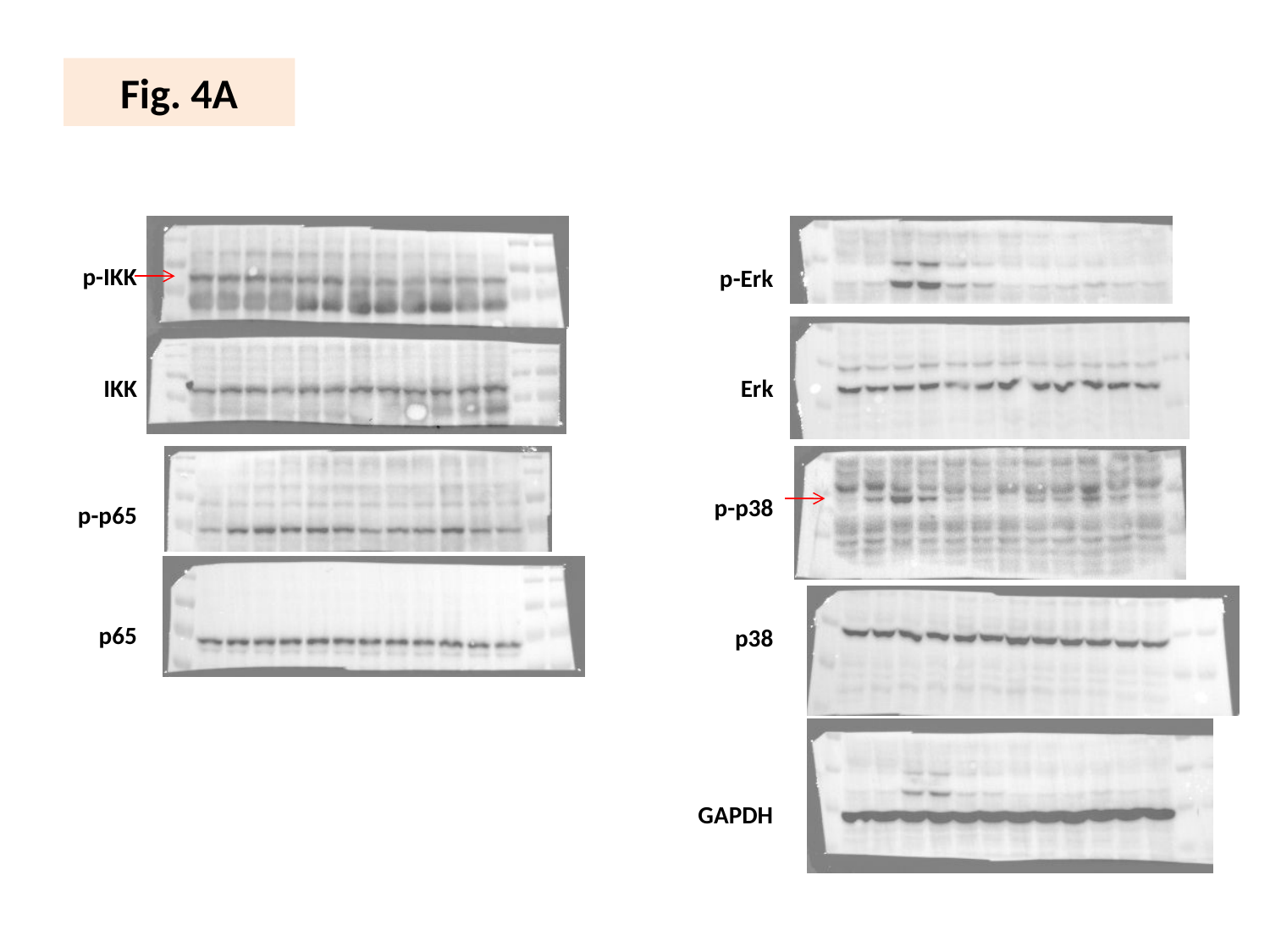

# Fig. 4A
p-IKK
p-Erk
IKK
Erk
p-p38
p-p65
p65
p38
GAPDH

## Slide 7
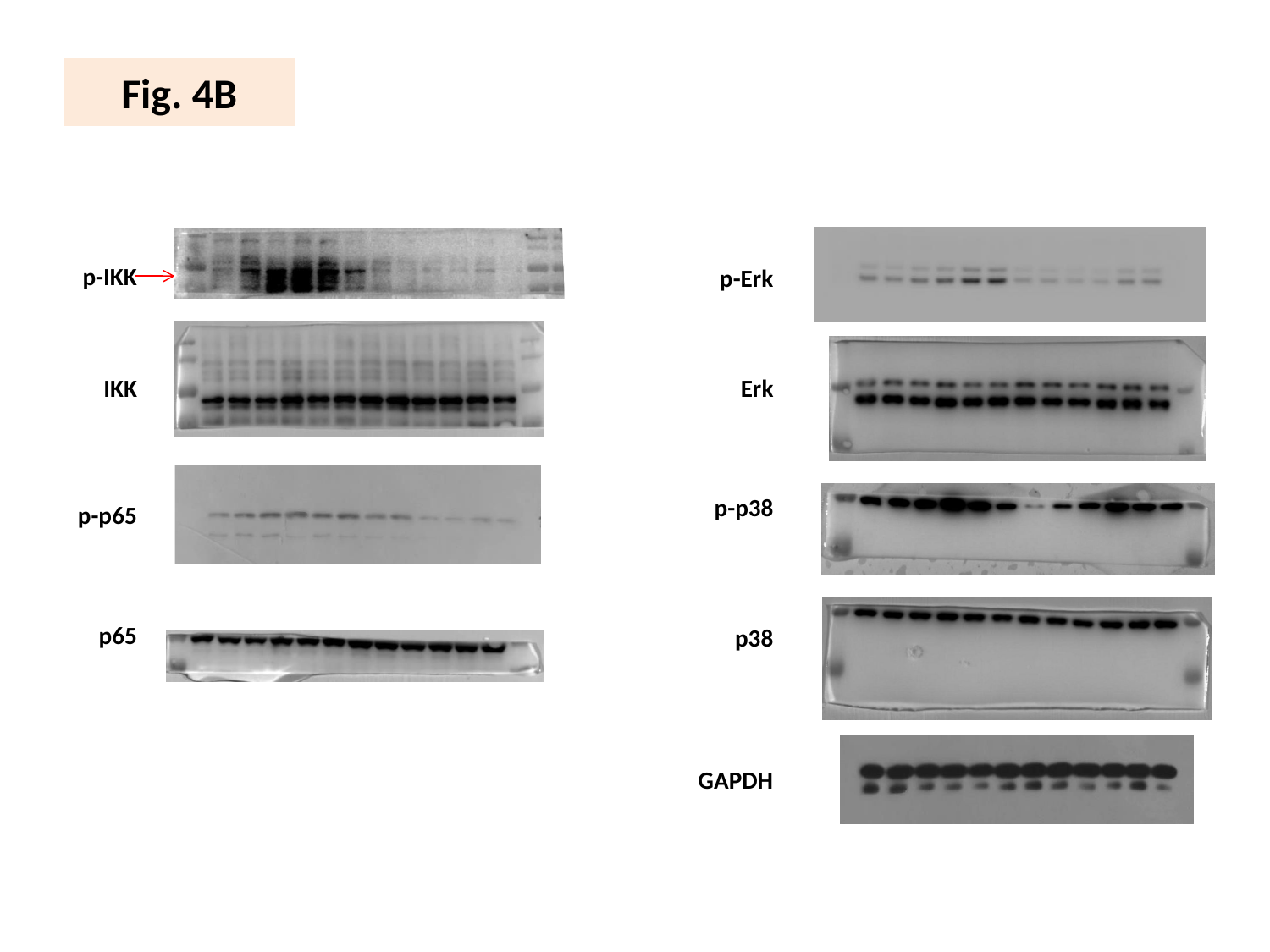

# Fig. 4B
p-IKK
p-Erk
IKK
Erk
p-p38
p-p65
p65
p38
GAPDH

## Slide 8
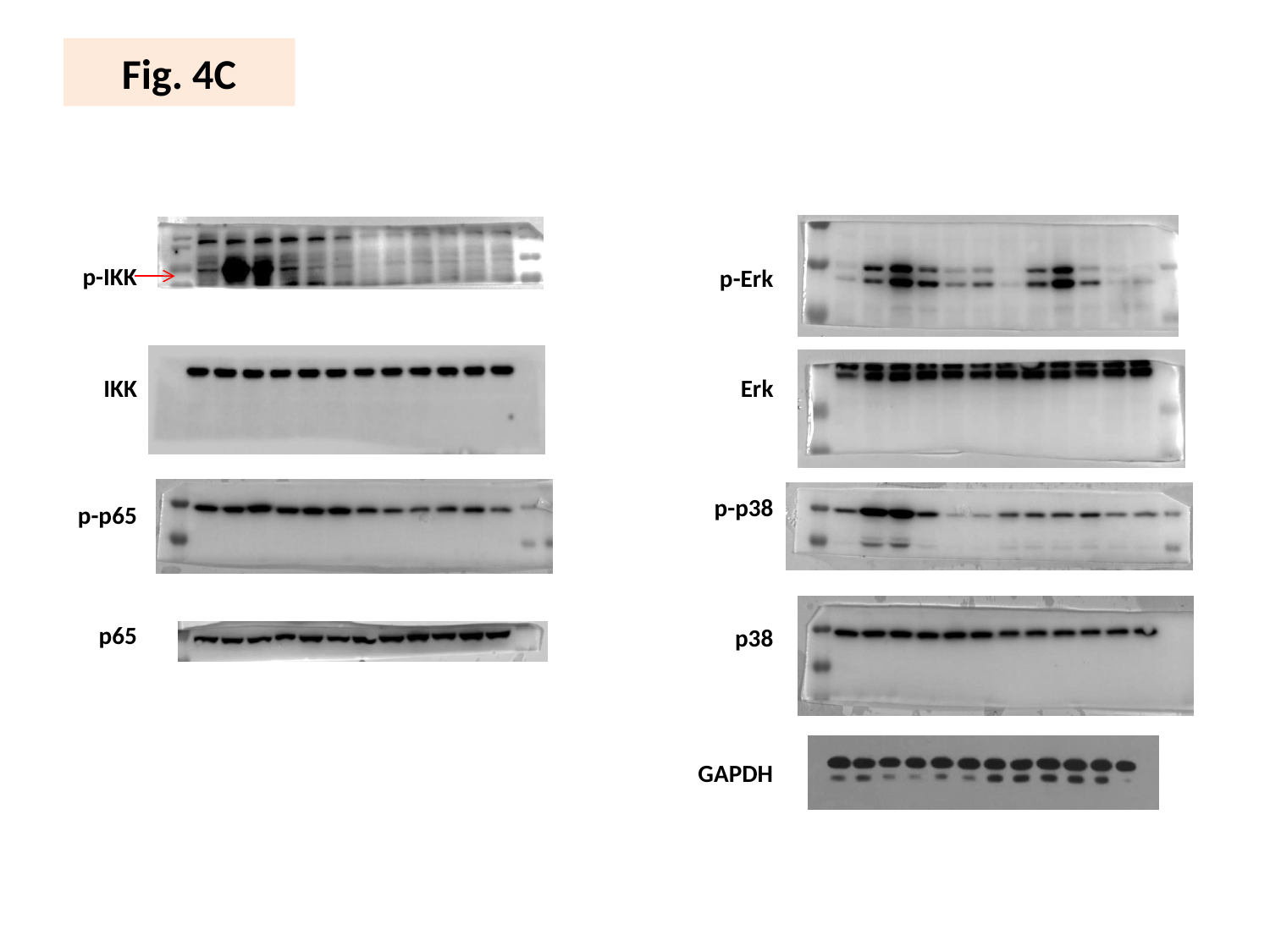

# Fig. 4C
p-IKK
p-Erk
IKK
Erk
p-p38
p-p65
p65
p38
GAPDH

## Slide 9
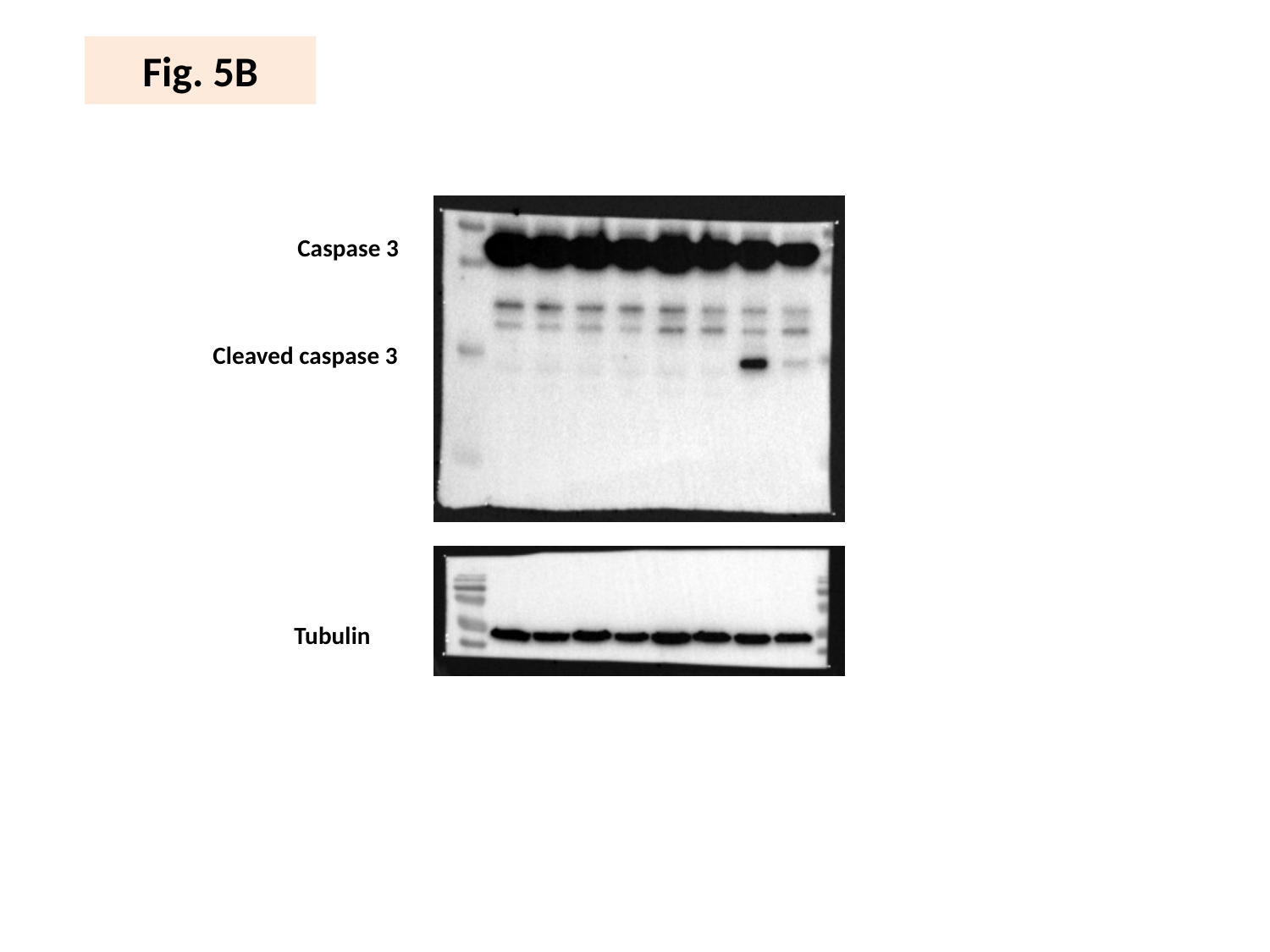

Fig. 5B
Caspase 3
Cleaved caspase 3
Tubulin

## Slide 10
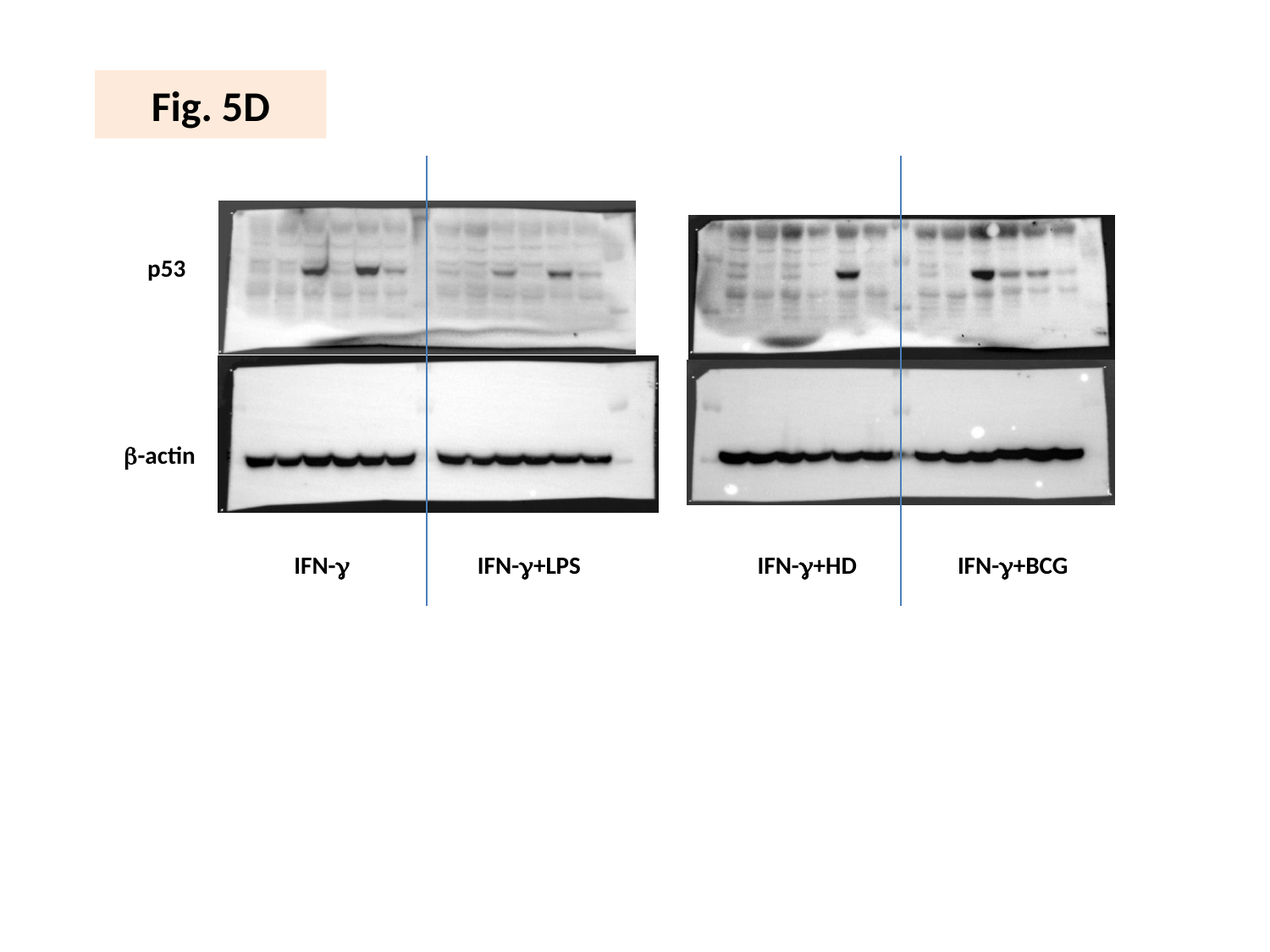

Fig. 5D
p53
b-actin
IFN-g
IFN-g+LPS
IFN-g+HD
IFN-g+BCG
